# Video-Assisted Endoscopic Laryngosurgery Using a Direct Laryngoscope and a Long Rigid Endoscope

**DOI:** 10.1155/DTE.6.51

**Published:** 2000

**Authors:** Masahiro Kawaida, Hiroyuki Fukuda, Naoyuki Kohno

**Affiliations:** 1 Department of Otolaryngology Tokyo Metropolitan Ohtsuka Hospital 2-8-1, Minamiohtuka Toshima-ku Tokyo 170-0005 Japan; 2 Department of Otolaryngology Keio University School of Medicine 35, Shinanomachi Shinjuku-ku Tokyo 160-0016 Japan; 3 Department of Otolaryngology National Defense Medical College 3-2, Namiki Tokorozawa-shi Saitama 359-0042 Japan

## Abstract

Endolaryngeal microscopic laryngosurgery (microlaryngosurgery) using a direct laryngoscope is the preferred surgical method for treating laryngeal lesions under general anesthesia. However, this method does not provide a wide-angle view of the larynx and does not allow detailed observations of the ventricle and subglottis of the laryngeal cavity, resulting in blind areas. Video-assisted endoscopic laryngosurgery using a direct laryngoscope and a long transurethral rigid endoscope was therefore utilized to allow clear observations and complete resection of laryngeal lesions in these blind areas. This endoscopic surgical technique is introduced, and clinical cases are presented.


*Diagnostic and Therapeutic Endoscopy*, Vol. 6, pp. 51–57
Reprints available directly from the publisher
Photocopying permitted by license only

© 2000 OPA (Overseas Publishers Association) N.V.
Published by license under
the Harwood Academic Publishers imprint,
part of The Gordon and Breach Publishing Group.
Printed in Malaysia.

### Video-Assisted Endoscopic Laryngosurgery Using a Direct Laryngoscope and a Long Rigid Endoscope

MASAHIRO KAWAIDA[a,*], HIROYUKI FUKUDA[b] and NAOYUKI KOHNO[c]

[a]*Department of Otolaryngology, Tokyo Metropolitan Ohtsuka Hospital, 2-8-1, Minamiohtuka, Toshima-ku, Tokyo 170-0005, Japan;* [b]*Department of Otolaryngology, Keio University School of Medicine, 35, Shinanomachi, Shinjuku-ku, Tokyo 160-0016, Japan;* [c]*Department of Otolaryngology, National Defense Medical College, 3-2, Namiki, Tokorozawa-shi, Saitama 359-0042, Japan*

*(Received 18 May 1999; In final form 15 July 1999)*

**Endolaryngeal microscopic laryngosurgery (microlaryngosurgery) using a direct laryngoscope is the preferred surgical method for treating laryngeal lesions under general anesthesia. However, this method does not provide a wide-angle view of the larynx and does not allow detailed observations of the ventricle and subglottis of the laryngeal cavity, resulting in blind areas. Video-assisted endoscopic laryngosurgery using a direct laryngoscope and a long transurethral rigid endoscope was therefore utilized to allow clear observations and complete resection of laryngeal lesions in these blind areas. This endoscopic surgical technique is introduced, and clinical cases are presented.**

*Keywords:* Direct laryngoscope, Endoscopic laryngosurgery, Long rigid endoscope, Microscopic laryngosurgery, Video system

### INTRODUCTION

Endolaryngeal microscopic laryngosurgery (microlaryngosurgery) using a direct laryngoscope is the preferred surgical method for treating laryngeal lesions. Although microscopic direct laryngoscopy (microlaryngoscopy) offers good visualization of the upper aspect of the laryngeal cavity, it does not provide a wide-angle view of the laryngeal cavity or allow detailed observations of the lateral side of the ventricle and subglottis, resulting in blind areas. These blind areas can cause difficulties when laryngeal lesions are being completely resected. Video-assisted endoscopic laryngosurgery using a direct laryngoscope and a long rigid endoscope was therefore utilized to resect lesions in these blind areas.

### MATERIALS AND METHODS

### Subjects

Three hundred and ten patients underwent endolaryngeal laryngosurgery using a direct laryngoscope

* Corresponding author. Tel.: +81-3-3941-3211. Fax: +81-3-3941-9557.

over a three-year period (January 1996 to December 1998) at the Tokyo Metropolitan Ohtsuka Hospital. Sixteen of these patients were treated with the endoscopic surgical technique described in this paper. Fourteen of the patients were male and two were female, and their ages ranged from 44 to 78 years. Specifically, three of the patients were treated for vocal fold polyps, one for vocal fold cyst, one for Reinke's edema, five for white lesions of the vocal folds, one for laryngeal papillomatosis, and five for laryngeal cancers. Two of these cases are described here: a 55-year-old man with supraglottic cancer (Patient 1) and a 59-year-old man with multiple papillomatosis of the larynx (Patient 2).

### Equipment

A Hopkins II forward-oblique rigid endoscope (Karl Storz GmbH and Company, Germany) was used. The inserted portion had an effective length of 300 mm, and the outer diameter was 4 mm. The angle of vision was 30° (Fig. 1A). Originally, this long endoscope was developed for use in the field of urology and for pediatric bronchoscopies. The endoscope was connected to a xenon light source (Karl Storz Xenon 300) with a light-guide cable. Videoendoscopic observations and recordings were performed using a 3 charge-coupled device (3CCD) color video camera (Karl Storz Tricam-SL) (Fig. 1B), a Super VHS video-tape recorder (Panasonic NV-FS900), a color video monitor (JVC AV-M21A1) and a color video printer (Sony UP-5200).

The laryngosurgeries were performed using a conventional direct laryngoscope (Nagashima Medical Instruments Company, Japan) with a left side tube through which a fiber optic light carrier was inserted to provide forward illumination (Fig. 1C). The rigid endoscope was also inserted through the side tube, which had an inner diameter of 4.8 mm. A surgical microscope (Wild M-690) was also used.

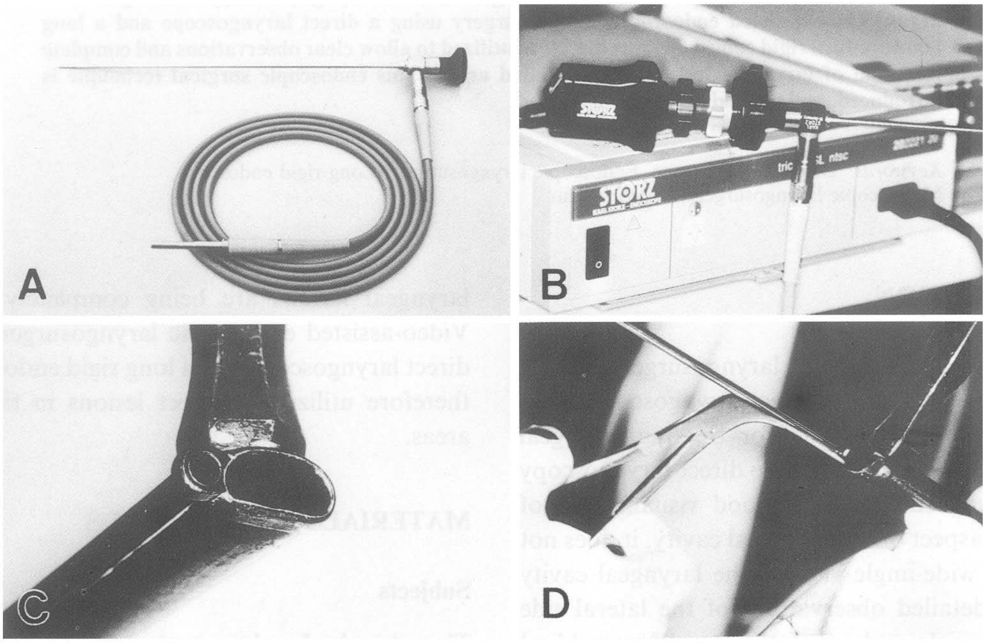

FIGURE 1 (A) Hopkins II 30° forward-oblique long rigid endoscope. (B) Karl Storz Tricam-SL 3CCD compact color video camera. (C) Nagashima direct laryngoscope with a left side tube for inserting a fiber optic light carrier. (D) A long rigid endoscope inserted in the side tube of a direct laryngoscope and the microsurgical forceps in the direct laryngoscope which are used to perform the laryngosurgery.

### Technique

After the patient was generally anesthetized using endotracheal intubation and inhalation, the direct laryngoscope was positioned and the laryngeal lesion was microscopically assessed. Videoendoscopic examinations were then performed using a 30° rigid endoscope connected to a 3CCD color video camera. The dynamic color images were projected onto the screen of the color video monitor. The laryngeal lesion was then resected using microlaryngeal forceps while observing the site of the lesion on the video monitor (Fig. 1D).

### RESULTS

Figure 2 shows the laryngeal cavity of Patient 1 as seen during microlaryngoscopy. Although an upper view of the laryngeal cavity was obtained, the tumor lesion in the supraglottis could not be clearly observed. The 30° forward-oblique rigid endoscope connected to the 3CCD color video camera was then inserted through the left side tube of the direct laryngoscope. This provided a wide-angle view of the entire supraglottic area and allowed the extent of the tumor on the anterior commissure of the ventricular folds to be seen (Fig. 3A). The lesion was then resected using microlaryngeal cutting forceps while videoendoscopically observing the site of the tumor (Fig. 3B).

In Patient 2, the straight-forward view of the laryngeal cavity was obtained with microlaryngoscopy. Only two papillomas on the posterior portion of the vocal folds could be seen during microlaryngoscopy (Fig. 4). When the entire laryngeal cavity was videoendoscopically observed using the 30° rigid endoscope, however, the wide-angle view of the larynx and the higher depth of field revealed the presence of five papillomas (Fig. 5A). All of the papillomas were then resected using microlaryngeal cutting forceps while videoendoscopically observing the site of lesions (Fig. 5B).

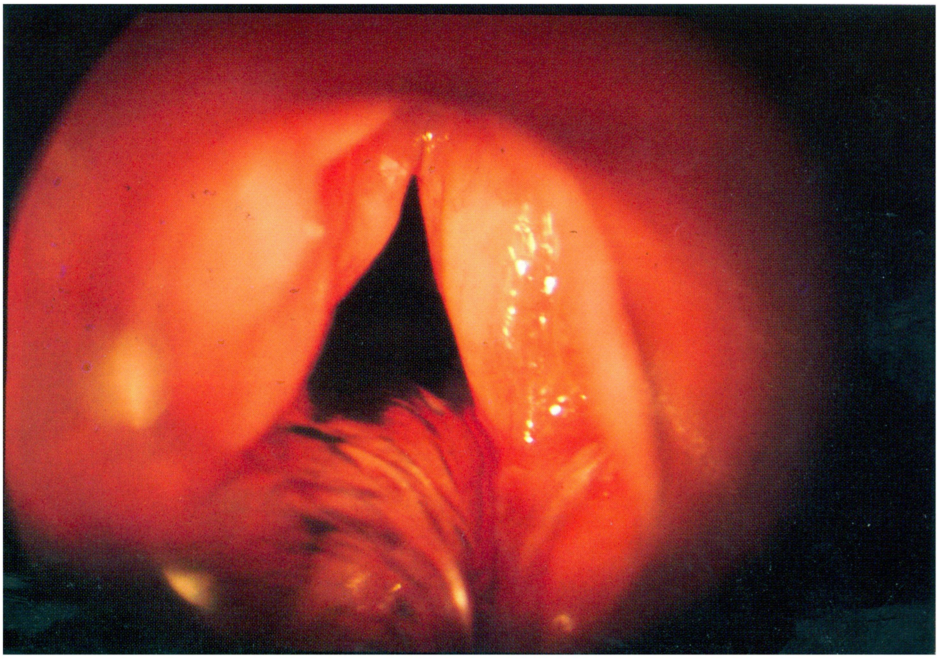

FIGURE 2 The laryngeal cavity as seen during microlaryngoscopy in Patient 1. The lesion on the supraglottis cannot be seen using a direct laryngoscope fitted with a surgical microscope.

A

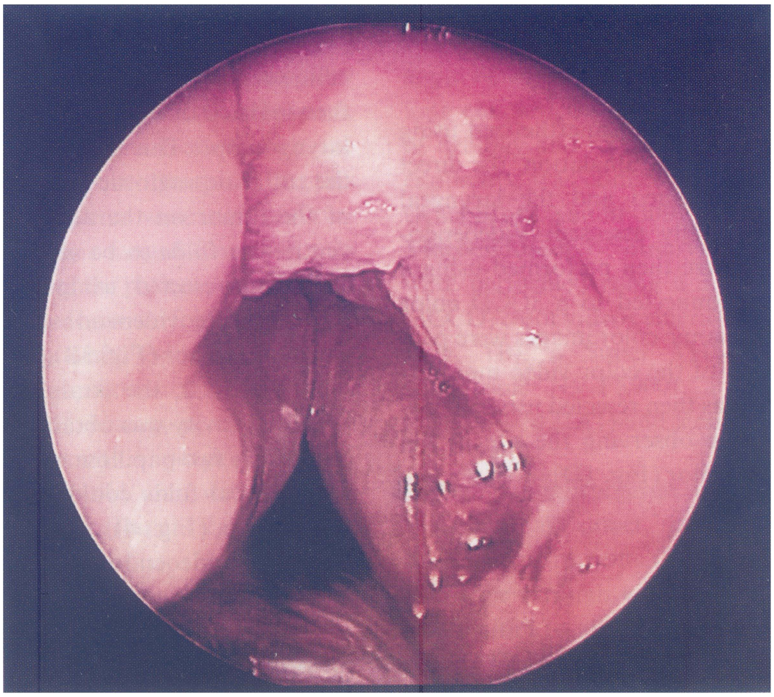

FIGURE 3A Videoendoscopic image of the tumor on the anterior commissure of the bilateral ventricular folds.

B

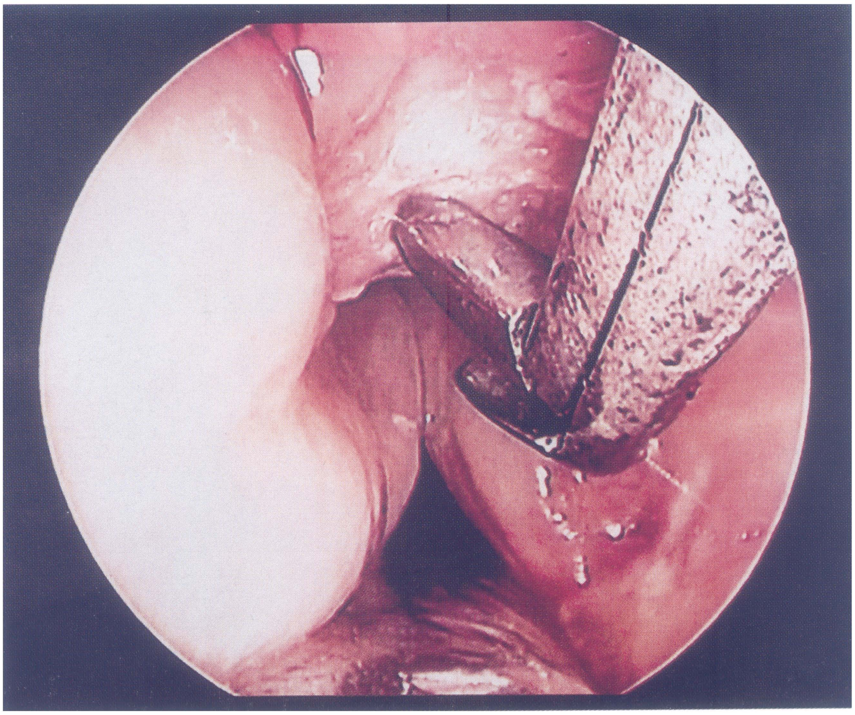

FIGURE 3B Resection of the tumor on the anterior commissure of the bilateral ventricular folds as seen during videoendoscopy.

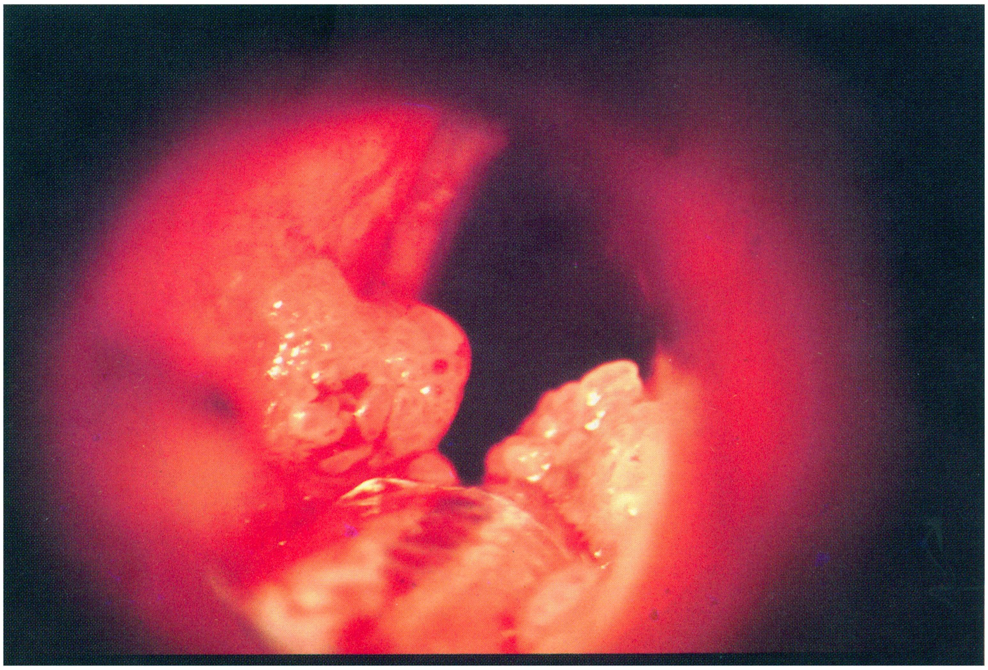

FIGURE 4 Image of two papillomas on the posterior portion of the vocal folds as seen using a direct laryngoscope fitted with a surgical microscope in Patient 2.

A

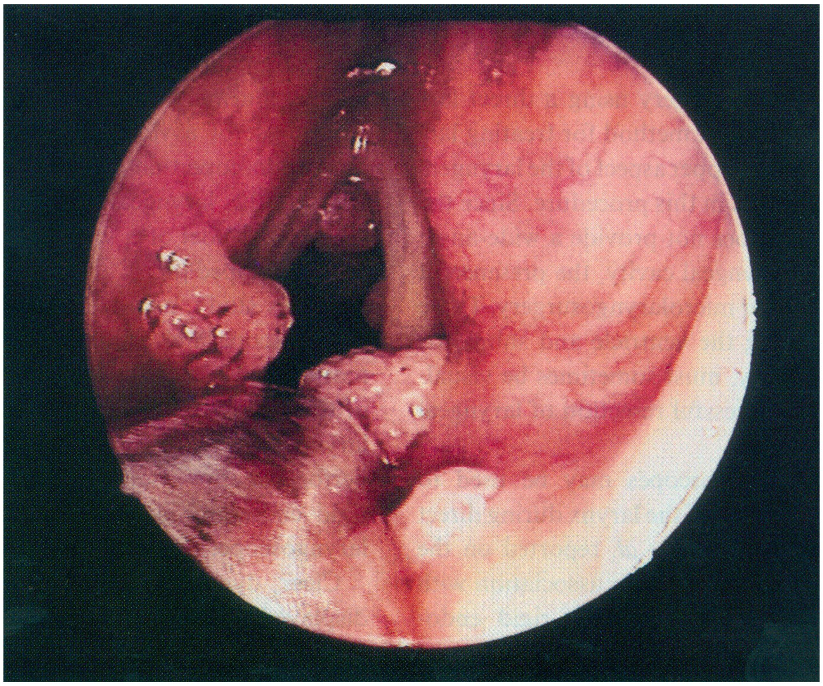

FIGURE 5A Videoendoscopic image revealing the presence of five papillomas.

B

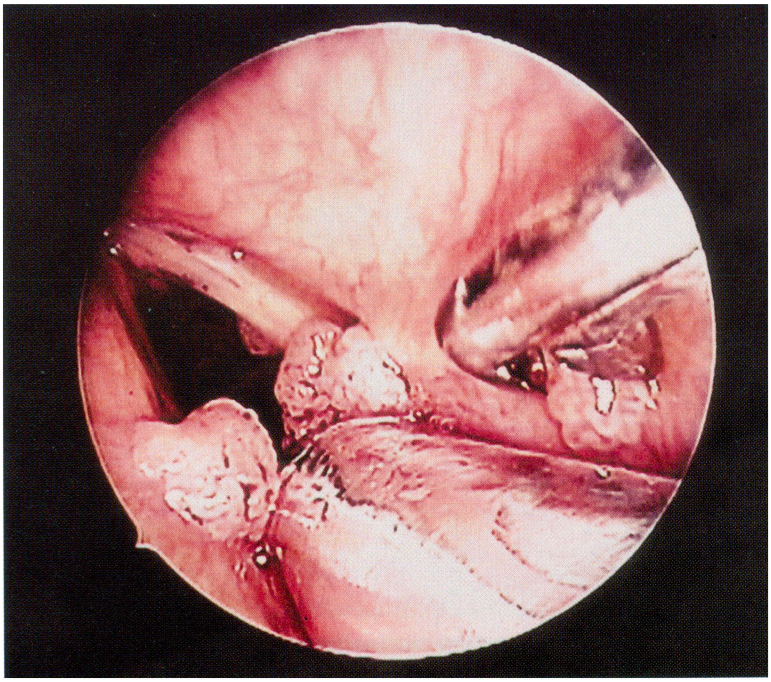

FIGURE 5B Resection of the papillomas using microlaryngeal cutting forceps as seen during videoendoscopy.

### DISCUSSION

Although microlaryngosurgery using a direct laryngoscope is the preferred method for treating laryngeal lesions, only the upper aspect of the laryngeal cavity can be visualized in this procedure. Since the direct laryngoscope does not provide a wide-angle upper view of the laryngeal cavity, the presence of blind areas may hinder microlaryngosurgery. These blind areas, including the ventricle and subglottis of the laryngeal cavity, must sometimes be visualized to ensure the successful resection of laryngeal lesions.

Special mirror-laryngoscopes may be used to observe these blind areas of the larynx during direct laryngoscopy [1,2]. Andrea *et al.* reported on the use of special rigid endoscopes in association with microlaryngeal surgery [3,4]. These rigid endoscopes had an outer diameter of 5 mm along the inserted portion and an effective length of 240 mm. Previously, we reported on observations of the endolarynx using long rigid endoscopes which were originally designed for the transurethral resection of urinary lesions [5]. Kantor *et al.* reported on the use of a video-laryngoscope containing a rigid endoscope that provided a wide-angle upper view of the laryngeal cavity [6,7]. Recently, an advanced model of a video-laryngoscope has also been developed [8].

In this study, a conventional Nagashima direct laryngoscope and a Hopkins II long rigid endoscope were used to resect lesions in the blind areas of the laryngeal cavity. Video-assisted endoscopic laryngosurgery using a direct laryngoscope was performed by inserting a 30° forward-oblique rigid endoscope into the side tube of the direct laryngoscope.

Clear, dynamic images of the blind areas were displayed on a color video monitor throughout the surgery. This enabled the surgeons to resect the lesions in the blind areas while observing magnified images of the area on the monitor. The long rigid endoscope was held securely in the side tube of the direct laryngoscope, providing stable images. The entire laryngeal cavity could be videoendoscopically visualized using the 30° forward-oblique rigid endoscope.

The surgical approach to the laryngeal cavity can be difficult if the patient has either a stiff neck or a large tongue. The technique presented in this study should make the surgical approach to the laryngeal cavity much easier in these cases.

### Acknowledgments

The authors wish to thank Mr. Takanori Sekioka (Karl Storz Endoscopy Japan K.K.), Mr. Susumu Ishiwata (Nippon Kayaku Co., Ltd.), Mr. Shigeru Enomoto (Nippon Kayaku Co., Ltd.) and Mr. Hiroyuki Matsuda (Schering-Plough K.K.) for their cooperations in this study.

### References

[1] G.L. Jako, Laryngoscopy for microscopic observation, surgery, and photography. *Arch. Otolaryngol.* 1970; **91**: 196–199.

[2] A. Shiotani, H. Fukuda, M. Kawaida *et al.* Newly designed specula for laryngomicroscopy. *Diagn. Ther. Endosc.* 1996; **3**: 73–78.

[3] M. Andrea, O. Dias and J. Paço. Endoscopic anatomy of the larynx. *Curr. Opin. Otolaryngol. Head Neck Surg.* 1994; **2**: 271–275.

[4] M. Andrea and O. Dias. *Atlas of Rigid and Contact Endoscopy in Microlaryngeal Surgery.* Philadelphia: Lippincott-Raven Publishers, 1995.

[5] M. Kawaida, H. Fukuda and N. Kohno. Multidirectional observations of the larynx using transurethral rigid endoscopes during direct laryngoscopy. *J. Laryngol. Otol.* 1998; **112**: 464–466.

[6] E.A. Kantor, G. Berci, E. Partlow *et al.* A completely new approach to microlaryngeal surgery. *Laryngoscope* 1991; **101**: 676–679.

[7] E.A. Kantor, G. Berci, E. Partlow *et al.* Ancillary instruments for the video microlarygoscope. *Ann. Otol. Rhinol. Laryngol.* 1991; **100**: 317–319.

[8] B. Benjamin. Thirty-five-millimeter photography using the Kantor–Berci video laryngoscope. *Ann. Otol. Rhinol. Laryngol.* 1998; **107**: 775–778.

